# Dalbavancin for Bone and Joint Infections: A Two-Center Greek Real-World Retrospective Study

**DOI:** 10.3390/pathogens14111109

**Published:** 2025-10-31

**Authors:** Christina Petropoulou, Petros Ioannou, Georgios Eleftherakis, Stefania Papazisi, Christos Davoulos, Eugenia Drosou, Anastasia Spiliopoulou, Ekaterini Tsiata, Fotini Paliogianni, Diamantis Kofteridis, Markos Marangos, Stelios F. Assimakopoulos

**Affiliations:** 1 Department of Internal Medicine, University Hospital of Patras, 26504 Patras, Greece; chripetr96@gmail.com (C.P.);; 2 Department of Internal Medicine, University Hospital of Heraklion, 71110 Heraklion, Greecestefaniapapazisi@gmail.com (S.P.);; 3 Department of Microbiology, University Hospital of Patras, 26504 Patras, Greece; 4 Department of Pharmacy, University Hospital of Patras, 26504 Patras, Greece

**Keywords:** dalbavancin, osteomyelitis, spondylodiscitis, septic arthritis, prosthetic joint infection, Gram-positive bacteria

## Abstract

Bone and joint infections remain therapeutic challenges, usually requiring prolonged intravenous therapy and hospitalization. Dalbavancin, a long-acting lipoglycopeptide, offers a simplified alternative. We retrospectively analysed 83 patients treated with dalbavancin for osteomyelitis, spondylodiscitis, septic arthritis, or prosthetic joint infection in two tertiary Greek hospitals (2022–2024). Mean age was 69 ± 16 years; 56.6% were male; Charlson Comorbidity Index averaged 4 ± 2.25. Common comorbidities included diabetes (28.9%) and coronary artery disease (18.1%). Infections were vertebral osteomyelitis/spondylodiscitis (48.2%), non-vertebral osteomyelitis (38.5%), prosthetic joint infection (10.8%), and septic arthritis (8.4%). Microbiological diagnosis was established in 62.6%; predominant pathogens were *Staphylococcus aureus* (38.4%: 30.7% MSSA, 7.7% MRSA) and enterococci (25%, including 5.7% VRE). Dalbavancin was administered as monotherapy (32.4%) or combined with other antibiotics (67.6%), mainly fluoroquinolones (63.6%) and minocycline (23.6%). Mean dosing was 2 ± 2.36 administrations (4 ± 4.38 g total). Surgical debridement was performed in 36.1% of patients. Clinically significant adverse events occurred in 4 patients (4.8%): acute kidney injury (*n* = 2), angioedema (*n* = 1), and *Clostridioides difficile* colitis (*n* = 1). Clinical cure was achieved in 90.4% at day 90 and 92.8% at day 180. Clinical cure rates were comparable between dalbavancin monotherapy and combination therapy, suggesting that the efficacy observed was primarily attributable to dalbavancin itself. Relapse at one year occurred in 10.8%, mainly due to inadequate source control. Dalbavancin demonstrated high efficacy, favourable safety, and treatment simplification in complex bone and joint infections. Its long half-life and reduced need for prolonged IV access support its role in minimizing hospitalization and catheter-related complications, particularly in regions with limited outpatient parenteral therapy infrastructure.

## 1. Introduction

Bone and joint infections, including osteomyelitis and spondylodiscitis, remain difficult-to-treat entities due to their chronicity, requirement for prolonged therapy, and frequent need for surgical management [1,2,3]. The incidence of osteomyelitis is estimated at 21.8 cases per 100,000 person-years, with a higher burden among elderly and diabetic patients [1]. Spondylodiscitis represents up to 4% of all osteomyelitis cases and carries significant morbidity and mortality [2]. Conventional intravenous antibiotic regimens typically extend for 6 to 12 weeks, necessitating prolonged hospitalizations or outpatient parenteral antimicrobial therapy (OPAT). This exposes patients to catheter-related complications, nosocomial infections, and adherence challenges [3]. In countries without established OPAT systems, such as Greece, these difficulties are magnified and often translate into worse patient outcomes and higher healthcare costs [4,5].

Dalbavancin is a novel lipoglycopeptide structurally related to vancomycin, with potent activity against methicillin-resistant *Staphylococcus aureus* (MRSA), methicillin-susceptible *S. aureus* (MSSA), streptococci, and susceptible enterococci [6,7]. Its pharmacologic properties include a long terminal half-life of 14.4 days [8], concentration-dependent bactericidal activity, and excellent tissue penetration, including into bone, as well as activity against biofilm-producing organisms [9]. These characteristics render it highly suitable for deep-seated and chronic Gram-positive infections. While originally approved for acute bacterial skin and skin structure infections (ABSSSIs), dalbavancin has been increasingly used off-label in osteomyelitis, prosthetic joint infections, and spondylodiscitis [7,10,11,12,13]. Over the past decade, a growing body of real-world studies, case series, and systematic reviews has demonstrated favourable outcomes, particularly with multidose regimens [11,12,13]. However, evidence remains heterogeneous, and randomized controlled trials are still lacking.

The aim of this study is to provide real word data on dalbavancin use for bone infections. The findings of the present study are particularly relevant in the setting of a healthcare system challenged by multidrug resistance and lacking structured OPAT infrastructure [4,5].

## 2. Materials and Methods

### 2.1. Study Population

This retrospective observational study was conducted at the University General Hospital of Patras (UGHP) and the University General Hospital of Heraklion (UGHH), Greece, between 1 January 2022 and 31 June 2024. Ethical approval was obtained from both institutions’ Ethics Committees (UGHP No: 325/13.07.2023 and UGHH No:844/12.08.2025). Informed consent was waived in accordance with institutional policies, since the study was observational and retrospective in nature and all patient data were anonymized according to GDPR standards.

Eligible patients were adults aged 18 years or older, with a documented diagnosis of bone and/or joint infection who received at least one dose of dalbavancin for this off-label indication. The diagnosis of osteomyelitis was based on the consensus criteria of the European Societies of Nuclear Medicine, Bone and Joint Infections, Radiology, and Clinical Microbiology and Infections [1]. The diagnosis of spondylodiscitis followed the 2015 Clinical Practice Guidelines of the Infectious Diseases Society of America [2]. The diagnosis of prosthetic joint infections was based on the 2019 IDSA/AAOS/ACR/SNMMI guidelines [3]. In culture-negative cases, the diagnosis was based on the combination of compatible clinical presentation, imaging findings (magnetic resonance imaging or bone scintigraphy), and elevated inflammatory markers, in accordance with the European and the Infectious Diseases Society of America consensus criteria referred above. In several such cases, diagnostic certainty was further supported by subsequent radiologic improvement and normalization of inflammatory markers following dalbavancin therapy.

The decision to administer dalbavancin as monotherapy or in combination with other antimicrobials was left to the discretion of the treating physicians, reflecting the off-label nature of use and absence of standardized protocols. The commonest dosage schemes of dalbavancin were the following: 1.5 g in weeks 1 and 3, or 1.5 g in weeks 1 and 2, or three doses of 1.5 g with a two-week interval between them.

### 2.2. Outcomes

Patients were followed for up to twelve months after discontinuation of dalbavancin therapy. Clinical outcome was evaluated at three, six and twelve months after the administration of the last dose. Clinical cure was defined as resolution of signs and symptoms of infection, as assessed by the treating physicians, along with normalization of inflammatory markers. Imaging follow-up included magnetic resonance imaging and bone scintigraphy and were performed when clinically indicated. Treatment failure was defined as persistence or recurrence of fever or elevated inflammatory markers, the need for reoperation, switch to another antibiotic regimen, and/or death attributable to the infection.

### 2.3. Data Collection

Data were extracted from electronic medical records, microbiology laboratory information systems, and patient charts. Collected variables included demographics, comorbidities, Charlson Comorbidity Index (CCI), type, site and etiology of infection, presence of foreign material, microbiological findings, surgical management, dalbavancin regimen, use of concomitant antibiotics, adverse events and clinical outcomes.

### 2.4. Microbiological Analyses

Pathogen identification was performed using the VITEK^®^2 system (bioMérieux, Marcy-l’ Étoile, France) [14]. Antimicrobial susceptibility testing was conducted in accordance with the EUCAST criteria [15]. The results were assessed according to EUCAST guidelines, with isolates categorized as susceptible (including susceptible, increased exposure) or resistant [16].

## 3. Results

Patients’ demographic characteristics, comorbidities, type and cause of infection, diagnostic methods, microbiological results, treatment and outcomes are presented in detail in Table 1.

Regarding the type of infection, spondylodiscitis was the most frequent diagnosis (48.2%). The lumbar spine was the most affected anatomical site (36.1%), followed by the thoracic (9.6%) and cervical spine (2.4%). Non-vertebral osteomyelitis accounted for 38.5% of cases, with the lower limbs involved in 19.3%, upper limbs in 8.4%, and other bones (clavicle, pelvis, ribs) in 10.8%. Prosthetic joint infections were diagnosed in 10.8% of cases and septic arthritis in 6%.

The presumed etiology of infection was hematogenous in 19.3%, secondary to skin ulcers in 16.9%, traumatic in 13.2%, and postoperative in 10.8%. No source could be identified in 39.8%. The presence of foreign material was documented in 20.5% of patients.

Baseline laboratory values reflected systemic inflammation: mean ESR was 75 ± 40 mm/h and mean CRP was 2.0 ± 2.7 mg/dL. The mean baseline WBC was 8000 ± 2200/mm^3^. Mean estimated GFR was 85 ± 22 mL/min, with four patients developing transient renal impairment during treatment.

Microbiological diagnosis was attempted in all cases with blood cultures, while samples of bone or synovial fluid for culture were taken in 42.2% of patients. Staphylococci were the predominant pathogens (57.6%), comprising methicillin-sensitive *S. aureus* (MSSA, 30.7%), methicillin-resistant *S. aureus* (MRSA, 7.6%), methicillin-sensitive coagulase-negative staphylococci (MSCNS, 17.3%), and methicillin-resistant coagulase-negative staphylococci (MRCNS, 1.9%). Streptococci accounted for 17.3% of isolates. Enterococci were recovered in 25% of cases, including *E. faecalis* (13.4%) and *E. faecium* (11.5%), with vancomycin-resistant enterococci (VRE) detected in 5.7%.

Among the 83 patients included, surgical debridement was performed in 30 (36.1%) cases, either followed by dalbavancin monotherapy (*n* = 9, 10.8%) or by dalbavancin in combination with other antimicrobial agents (*n* = 21, 25.3%). In patients who did not undergo debridement (*n* = 53, 63.9%), dalbavancin was administered as monotherapy in 18 (21.6%) and as part of combination therapy in 35 (42.1%). The mean number of dalbavancin doses administered was 2 ± 2.36, corresponding to a cumulative dose of 4 ± 4.38 g. The commonest dosage schemes were the following: 24% received 1.5 g in weeks 1 and 3 and 21% received 1.5 g in weeks 1 and 2, 10% received three doses of 1.5 g with a two-week interval between them.

Overall, 56 patients (67.5%) received dalbavancin in combination with other agents. Combination therapy was applied in 13 of 16 culture-negative cases (81%) and in 43 of 67 culture-positive cases (64.1%). The most frequent companion antibiotics were fluoroquinolones (62.5%), followed by minocycline (23.6%), β-lactams (19.6%), trimethoprim-sulfamethoxazole (12.5%), rifampicin (7.1%), linezolid (5.3%), and daptomycin (3.6%).

Clinically significant adverse events were observed in 4 patients (4.6%). Two patients developed acute kidney injury during treatment, which resolved gradually without the need for renal replacement therapy, one patient developed angioedema successfully treated with corticosteroids and one patient CDI colitis treated with oral vancomycin. The length of hospitalization was 19 ± 25.4 days. Clinical outcomes were favorable, with cure achieved in 75 patients (90.4%) at day 90 and in 77 patients (92.8%) at day 180. At one-year follow-up, relapse was documented in 9 cases (10.8%). Of these, seven (77%) involved vertebral osteomyelitis or spondylodiscitis, none had prosthetic material, and only three (33%) underwent surgical debridement. In addition, among the nine patients who experienced relapse, three received dalbavancin 1.5 g in weeks 1 and 2, while six received 1.5 g every two weeks, with treatment duration varying substantially. Given this marked heterogeneity and the small number of relapsing cases, no meaningful association can be established between a specific dosing regimen and relapse risk. The same limitation applies to the microbiological findings, as diverse pathogens were identified in seven of the nine relapsing patients. Clinical outcomes were comparable between patients treated with dalbavancin monotherapy and those receiving combination regimens. Relapse occurred in 3/27 (11.1%) of monotherapy patients and in 6/56 (10.7%) of combination therapy patients. In monotherapy, clinical cure at day 90 was achieved in 88.9% and at day 180 in 92.6%, and in combination therapy in 91.1%, and 92.9% of patients, respectively.

## 4. Discussion

This study demonstrates that dalbavancin is a safe and effective therapeutic option for bone and/or joint infections, achieving cure rates of over 90% at 6 months. These findings are highly encouraging when considered in the context of the Greek healthcare system, which is characterized by a high prevalence of multidrug-resistant organisms and an absence of a structured OPAT network [4,5,10]. Under such conditions, prolonged inpatient IV therapy carries significant risks, including nosocomial infections, vascular catheter complications, and increased healthcare costs. Dalbavancin offers a valuable alternative by facilitating early discharge, reducing exposure to hospital-acquired infections, and simplifying treatment logistics. Although dalbavancin was principally used for early discharge, the mean hospital stay in our cohort (19 ± 25.4 days) is relatively high but consistent with previous reports in spondylodiskitis [17]. This likely reflects the complex nature of our cases, particularly the need for surgical interventions and the high rate of multiple comorbidities, which would inherently prolong hospitalization even in optimized antimicrobial strategies.

A previous study by Tobudic et al. described outcomes in patients with osteomyelitis, spondylodiscitis, septic arthritis, and prosthetic joint infections, reporting cure rates of 64% with dalbavancin monotherapy, with failures associated with diabetes, MRSA infection, and inability to achieve source control [7]. Lovatti et al. conducted a systematic review of 450 patients and found an overall success rate of 79.3%, increasing to 92.3% when at least three 1500 mg doses were used, highlighting both efficacy and the potential role of optimized dosing regimens [11]. Similarly, Parruti et al. reported success rates of 84–91% in bone and prosthetic infections, with improved outcomes in patients receiving higher initial loading doses and undergoing surgical debridement [12]. Morata et al. confirmed the safety and efficacy of prolonged dalbavancin therapy in bone and implant-related infections, including cases without foreign body removal, when administered as repeated biweekly 1500 mg doses [13]. In a comparative cohort study, Cain et al. demonstrated that dalbavancin achieved outcomes equivalent to standard-of-care regimens in osteomyelitis, while significantly reducing length of stay and catheter-related complications [17]. Meta-analytical evidence by Almangour et al. further confirmed the effectiveness of multi-dose dalbavancin regimens in osteomyelitis, with outcomes comparable to conventional therapy but with fewer adverse events and shorter hospitalization [18]. Reports of high success rates in spinal infections, including spondylodiscitis (85–89%) [19,20], add further support for its role in difficult-to-treat skeletal infections.

Pharmacologically, dalbavancin’s long half-life, antibiofilm activity, and excellent tissue penetration support its use in bone infections [8,9]. Unlike vancomycin, which requires frequent monitoring and carries nephrotoxicity risks, or daptomycin, which is associated with myopathy, dalbavancin combines potent Gram-Positive activity with superior tolerability and ease of administration [6,7]. With respect to safety, clinically significant adverse events occurred in 4.8% of our patients, a rate that is generally lower than those reported in previous cohorts, underscoring dalbavancin’s favorable safety profile even with multi-dose regimens [13]. This relatively low adverse event rate may reflect the shorter overall antibiotic exposure, limited use of concomitant nephrotoxic drugs, and the close post-discharge follow-up that enabled timely identification and management of treatment-related events. The drug’s favorable safety profile, combined with flexible dosing schemes, allows for individualized treatment approaches, particularly valuable in complex patients.

An additional consideration in Greece and similar epidemiological contexts is the potential of dalbavancin to reduce the risk of nosocomial infections. By facilitating earlier discharge and minimizing inpatient exposure, dalbavancin indirectly limits patient contact with multidrug-resistant Gram-Negative organisms, which are endemic in Greek hospitals [4,5,10]. This collateral benefit is particularly relevant in overburdened health systems. Beyond the individual patient-level outcomes, dalbavancin has significant health-system and socioeconomic implications. Shorter hospitalization translates into improved bed availability, which is critical in tertiary hospitals constantly under pressure from overcrowding. Furthermore, by minimizing the use of central venous catheters, dalbavancin reduces the risk of line-associated bloodstream infections and thrombosis, complications that not only worsen prognosis but also further increase healthcare costs [21]. In addition, the ability to simplify therapy and allow patients to continue treatment in the community enhances quality of life, particularly for elderly individuals and those with limited mobility. These aspects have been increasingly recognized in pharmaco-economic analyses, and our data strongly support such perspectives in real-world practice.

Another key aspect is the role of dalbavancin in the broader strategy of antimicrobial stewardship. Its convenient dosing, combined with demonstrated efficacy, offers a tool to optimize antibiotic use, especially in high-resistance settings where alternative options are limited [13,17]. Infections caused by MRSA, VRE, or biofilm-associated organisms often require prolonged therapy, where toxicity or adherence issues may compromise outcomes. Dalbavancin provides an option with fewer adverse effects and reduced monitoring needs, which is particularly relevant for resource-limited healthcare systems.

It is also important to recognize the potential limitations of dalbavancin. While our study and others highlight its strong efficacy profile, the heterogeneity of dosing regimens across real-world series prevents definitive conclusions about the optimal regimen. In our cohort, the most common dosage schemes included two 1.5 g doses given in weeks 1 and 3 (24%) or in weeks 1 and 2 (21%), while 10% of patients received three 1.5 g doses at two-week intervals. Although two doses of 1500 mg one week apart appear to be the most consistently successful approach in prior series, our data suggest that multiple alternative regimens are applied in clinical practice. Notably, the 10.8% relapse rate in our cohort was largely observed among patients with vertebral osteomyelitis–spondylodiscitis (77%), all without prosthetic material and with limited surgical debridement (33%). These observations highlight that vertebral osteomyelitis is a deep-seated infection where effective surgical source control is often difficult to achieve but remains crucial for definitive treatment success.

The potential role of combination therapy warrants further consideration. In our cohort, concomitant antibiotics were administered in more than two-thirds of patients, most frequently fluoroquinolones or beta-lactams. Notably, combination therapy was used in 64.1% of culture-positive cases—likely to enhance biofilm penetration or activity against specific pathogens—and in 81% of culture-negative cases, to ensure empirical coverage for possible Gram-Negative or polymicrobial involvement. In real-world clinical practice, the isolation of a single pathogen does not necessarily exclude mixed infections, while low-virulence skin commensals such as coagulase-negative staphylococci may raise uncertainty regarding their etiologic role, prompting clinicians to maintain an additional companion agent. Moreover, in the absence of validated national protocols and given the off-label nature of dalbavancin use for bone and joint infections, physicians often adopted combination regimens as a pragmatic safety measure. These treatment patterns mirror broader real-world experience, where multi-agent strategies are commonly employed to address diagnostic uncertainty or biofilm-related infections. Whether dalbavancin can ultimately replace or reduce the need for such combinations remains an important clinical question, with implications for treatment simplification, antimicrobial stewardship, and reduction in adverse events. In our cohort, clinical outcomes were comparable between dalbavancin monotherapy and combination therapy, suggesting that the efficacy observed in bone and joint infections was primarily attributable to dalbavancin itself.

The present study is limited by its retrospective, descriptive design, absence of a control group, and heterogeneity in dosing and combination regimens, reflecting real-world, off-label clinical use. As such, causal inferences regarding the efficacy of dalbavancin monotherapy versus combination therapy cannot be definitively drawn.

In conclusion, dalbavancin represents a pivotal addition to the therapeutic armamentarium against bone and/or joint infections. Its efficacy, safety, and simplicity are particularly advantageous in high-MDR, OPAT-limited settings such as Greece. Strengthening the evidence base with prospective, controlled, and pharmacoeconomic studies will be crucial to defining its precise role, but the existing data, including the results of the present study, clearly demonstrate its potential to transform the management of these complex infections.

## Figures and Tables

**Table 1:** Clinical characteristics of patients treated with dalbavancin for bone and/or joint infections.

Parameter	Value	(%)
**Νumber of patients**	83	
**Gender** (male)	47	56.6%
**Age** (mean, SD)	69 ± 16.07	
**Length of Hospitalization** (days) (mean ± SD)	19 ± 25.48	
**Comorbidities**		
Diabetes Mellitus	24	28.9%
Coronary Arteries Disease	15	18.1%
COPD	6	7.2%
Peripheral Artery Disease	5	6%
Connective Tissue Disease	5	6%
Hematologic malignancy	5	6%
Dementia	4	4.8%
Immunosuppressive treatment	4	4.8%
Solid tumor malignancy	4	4.8%
Congestive Heart Failure	3	3.6%
Chronic Kidney Disease	3	3.6%
Chronic Liver Disease	2	2.4%
Stroke	1	1.2%
HIV	0	0%
Charlson Comorbidity Index (mean, SD)	4 ± 2.25	
**Type of Infection**		
*Non-vertebral Osteomyelitis*	32	38.5%
- Upper Limb	7	8.4%
- Lower Limb	16	19.3%
- Other site	9	10.8%
*Vertebral osteomyelitis—Spondylodiscitis*	40	48.2%
- Cervical Spine	2	2.4%
- Thoracic Spine	8	9.6%
- Lumbar Spine	30	36.1%
*Prosthetic Joint Infection*	9	10.8%
*Septic Arthritis*	5	6%
**Cause**		
Postoperative	9	10.8%
Skin ulcer	14	16.9%
Injury	11	13.2%
Hematogenous	16	19.3%
Unknown	33	39.8%
Foreign Material	17	20.5%
**Laboratory Values**		
Baseline WBC/mm^3^ (mean, SD)	8000 ± 2200	
Baseline ESR mm/h (mean, SD)	75 ± 40.15	
Baseline CRP mg/dL (mean, SD)	2 ± 2.68	
Baseline GFR mL/min (mean, SD)	85 ± 21.80	
**Diagnostic Methods for Microbiological Diagnosis**		
Blood culture	83	100%
Bone/tissue/synovial biopsy/culture	35	42.2%
Positive culture	52	62.6%
Negative culture	16	19.3%
**Microbiology**		
Staphylococci	30	57.6%
- MSSA	16	30.7%
- MRSA	4	7.7%
- MSCNS	9	17.3%
- MRCNS	1	1.9%
Streptococci	9	17.3%
Enterococci	13	25%
- *E. faecalis*	7	13.4%
- *E. faecium*	6	11.5%
- VRE	3	5.7%
**Treatment—Pharmacologic regimen**		
Surgical Debridement + dalbavancin monotherapy	9	10.8%
Surgical Debridement + dalbavancin + other(s) antibiotics	21	25.3%
Dalbavancin monotherapy (w/o surgical debridement)	18	21.6%
Dalbavancin + other(s) antibiotics (w/o surgical debridement)	35	42.1%
Dalbavancin number of doses (mean ± SD)	2 ± 2.36	
Total grams of Dalbavancin (mean ± SD)	4 ± 4.38	
**Combination treatment according to culture results**		
Combination therapy in culture negative cases	13/16	81%
Combination therapy in culture positive cases	43/67	64.1%
**Companion antibiotics**		
Fluoroquinolones	35	62.5%
Minocycline	13	23.6%
Beta-Lactams	11	19.6%
TMP/SMX	7	12.5%
Rifampicin	4	7.1%
Linezolid	3	5.3%
Daptomycin	2	3.6%
**Clinically significant adverse events**	4	4.8%
Acute kidney injury	2	2.4%
Angioedema	1	1.2%
CDI infection	1	1.2%
**Length of Hospitalization** (days) (mean ± SD)	19 ± 25.4	
**Outcomes all patients**		
Clinical cure at day 90	75	90.4%
Clinical cure at day 180	77	92.8%
Relapse at 1 year	9	10.8%
**Outcomes Dalbavancin monotherapy**		
Clinical cure at day 90	24	88.9%
Clinical cure at day 180	25	92.6%
Relapse at 1 year	3	11.1%
**Outcomes Dalbavancin combination therapy**		
Clinical cure at day 90	51	91.1%
Clinical cure at day 180	52	92.9%
Relapse at 1 year	6	10.7%

Eighty-three patients were included. The cohort was predominantly male (56.6%), with a mean age of 69 ± 16 years. The mean Charlson Comorbidity Index was 4 ± 2.3, indicating a high burden of comorbidity. The most common comorbidities were diabetes mellitus (28.9%), coronary artery disease (18.1%), COPD (7.2%), connective tissue disease (6%), malignancy (10.8%), and chronic kidney disease (3.6%).

## Data Availability

The data generated and analyzed during the current study were retrieved from the hospitals’ archives and the electronic patient record system for laboratory results. Due to patient privacy and ethical considerations, these data are not publicly available. Access may be granted upon request to the corresponding author, subject to approval by the relevant institutional review boards.

## Abbreviations

The following abbreviations are used in this manuscript:

Abbreviation	Full Form
ABSSSIs	Acute Bacterial Skin and Skin Structure Infections
ACR	American College of Rheumatology
AAOS	American Academy of Orthopaedic Surgeons
CDI	*Clostridioides difficile* Infection
CCI	Charlson Comorbidity Index
CDC	Centers for Disease Control and Prevention
COPD	Chronic Obstructive Pulmonary Disease
CRP	C-Reactive Protein
ECDC	European Centre for Disease Prevention and Control
ESR	Erythrocyte Sedimentation Rate
EUCAST	European Committee on Antimicrobial Susceptibility Testing
GDPR	General Data Protection Regulation
GFR	Glomerular Filtration Rate
HIV	Human Immunodeficiency Virus
IDSA	Infectious Diseases Society of America
IV	Intravenous
MRSA	Methicillin-Resistant *Staphylococcus aureus*
MRCNS	Methicillin-Resistant Coagulase-Negative Staphylococci
MSCNS	Methicillin-Susceptible Coagulase-Negative Staphylococci
MSSA	Methicillin-Susceptible *Staphylococcus aureus*
OPAT	Outpatient Parenteral Antimicrobial Therapy
SD	Standard Deviation
SNMMI	Society of Nuclear Medicine and Molecular Imaging
TMP/SMX	Trimethoprim/Sulfamethoxazole
VRE	Vancomycin-Resistant Enterococci
WBC	White Blood Cell count
